# Complete and draft genome sequences of clinical *Mycobacterium tuberculosis* isolates from the Philippines

**DOI:** 10.1128/mra.00305-25

**Published:** 2025-06-10

**Authors:** Saul M. Rojas, Ma. Angelica A. Tujan, Angela Kae C. Valencia, Jessel Babe G. Capin, Timothy John R. Dizon, Ma. Cecilia G. Ama, Ramon P. Basilio, Dodge R. Lim, Lorenzo T. Reyes, Jody Phelan, Susana Campino, Taane G. Clark, Mari Rose A. De Los Reyes

**Affiliations:** 1 Advanced Molecular Technologies Laboratory (formerly Molecular Biology Laboratory), Laboratory Research Division, Research Institute for Tropical Medicine, Alabang, Philippines; 2 National Tuberculosis Reference Laboratory, Laboratory Research Division, Research Institute for Tropical Medicine, Alabang, Philippines; 3 Faculty of Infectious and Tropical Diseases, London School of Hygiene and Tropical Medicine, London, United Kingdom; 4 Faculty of Epidemiology and Population Health, London School of Hygiene and Tropical Medicine, London, United Kingdom; 5 Medical Department, Clinical Research Division, Research Institute for Tropical Medicine, Alabang, Philippines; Queens College Department of Biology, Queens, New York, USA

**Keywords:** *Mycobacterium tuberculosis*

## Abstract

This study reports the complete and draft genomes of 71 *Mycobacterium tuberculosis* isolates from the Philippines. Hybrid assembly using Illumina and Nanopore sequencing produced six complete genomes, while others had 2–422 contigs. These data enhance understanding of *M. tuberculosis* diversity and drug resistance, supporting targeted treatment strategies.

## ANNOUNCEMENT

Tuberculosis (TB) remains a major public health challenge in the Philippines, with an alarming 739,000 new cases reported in 2023 and a high incidence rate of 643 per 100,000 people. The situation is further exacerbated by the growing burden of drug-resistant TB and risk factors such as undernutrition, smoking, and HIV, which hinder effective control efforts (1). Whole-genome sequencing serves as a powerful tool for TB diagnosis, strain characterization, and drug resistance profiling, providing critical insights to inform treatment strategies and curb the spread of TB in the Philippines (2).

Study participants were recruited from various TB-DOTS Centers in Muntinlupa City, Philippines, between 2019 and 2021. This study was approved by the Research Institute for Tropical Medicine Institutional Review Board (IRB Protocol No. 2018-26). Sputum specimens were processed at the National Tuberculosis Reference Laboratory using the NALC-NaOH method, cultured in BD BACTEC MGIT tubes, and subcultured on Ogawa medium incubated at 37°C for up to 8 weeks. DNA was extracted via a phenol-chloroform protocol, which included heat inactivation, sequential incubations with lysozyme, 10% SDS, proteinase K, and 10% cetyltrimethylammonium bromide, followed by protein/lipid extraction with phenol-chloroform-isoamyl alcohol, DNA precipitation with isopropanol, and a final wash with 70% ethanol. DNA purity and concentration were assessed using a DS-11FX spectrophotometer (DeNovix Inc., USA) and a Qubit 3.0 fluorometer (Invitrogen, USA). Short-read sequencing libraries were prepared using the Illumina DNA Prep Kit (Illumina, USA) and sequenced on an Illumina MiSeq platform to generate 300 bp paired-end reads. Long-read libraries were constructed using the Rapid Barcoding Kit (SQK-RBK110.96) and sequenced on a MinION Mk1B (Oxford Nanopore Technology) using an R9.4.1 flow cell (FLO-MIN106D). No size selection or shearing was performed before library preparation. Base-calling and demultiplexing of Nanopore reads were performed using Guppy (v6.5.7), with read trimming carried out using Filtlong (v0.2.1) using the parameters “–min_length 1000” and “–min_mean_q 85” (https://github.com/rrwick/Filtlong) for Nanopore data and fastp (v0.23.4) (3) for Illumina data.

A long-read-first assembly strategy was employed following an established protocol (4). Initial assemblies were generated using Flye (v2.9.4) (5) and polished with Medaka (v1.11.2; https://github.com/nanoporetech/medaka). Subsequently, short-read polishing was performed using Polypolish (v0.6.0) (6) and POLCA (7). Assembly quality was assessed with QUAST (v5.2.0) (8). Drug resistance profiles and lineage assignments were determined using TB-Profiler (v6.2.1) (9). Default parameters were used unless otherwise noted.

The assembled genomes varied in length from 4.02 to 4.46 Mb, with GC content ranging from 65.24% to 65.63%. N50 values spanned 27,614–4,448,271 bp, with six samples yielding closed genomes, while others consisted of 2–422 contigs. Combined sequencing coverage ranged from 20.4- to 232.2-fold.

Based on *in silico* predictions, 24 samples were drug-sensitive, 26 were multidrug-resistant TB (MDR-TB), 8 were pre-MDR, 4 were rifampicin-resistant TB (RR-TB), and 9 had other resistance profiles. Lineage analysis revealed that eight samples belonged to the Euro-American lineage, while the rest were Indo-Oceanic. These findings provide insights into the *Mycobacterium tuberculosis* strains in Muntinlupa City, supporting infection control efforts in the Philippines.

**TABLE 1:** Assembly details and GenBank accession numbers of *M. tuberculosis* clinical strains from the Philippines

Illumina sequencing			ONT sequencing				GenBank assembly accession no.	Genome size (bp)	No. of contigs	Assembly N_50_ (bp)	GC content (%)	No. of CDS	Lineage^*a*^	Drug resistance type^*b*^
SRA accession no.	No. of reads	Coverage depth (×)	SRA accession no.	Read N_50_ (bp)	No. of reads	Coverage depth (×)								
SRR29081046	1,284,670	52	SRR29081119	3,506	388,917	180.4	CP157900	4,445,673	1	4,445,673	65.6	4,224	L1	Sensitive
SRR29081045	1,867,088	86	SRR29081118	3,263	280,889	123	CP157901	4,448,271	1	4,448,271	65.6	4,235	L1	Sensitive
SRR29081034	2,239,444	77	SRR29081107	3,536	313,154	143.5	JBEFMH000000000	4,448,080	3	4,443,925	65.59	4,238	L1	Sensitive
SRR29081023	972,956	61	SRR29081096	2,603	88,416	30.1	JBEFMI000000000	4,447,890	7	4,432,553	65.6	4,247	L1	Sensitive
SRR29081012	433,812	27	SRR29081085	3,540	102,310	47.3	JBEFMJ000000000	4,425,342	4	4,415,044	65.63	4,221	L4	HR-TB
SRR29080996	627,034	36	SRR29081074	2,966	106,864	43.2	JBEFMK000000000	4,463,997	9	4,426,861	65.6	4,255	L1	Sensitive
SRR29080985	482,614	29	SRR29081063	2,383	154,266	49.9	JBEFML000000000	4,442,840	4	4,431,240	65.59	4,241	L1	Sensitive
SRR29080974	57,088	3	SRR29081052	2,597	86,286	30.6	JBEFMM000000000	4,450,971	4	4,444,104	65.6	4,430	L1	Other
SRR29081007	1,186,802	50	SRR29081048	4,058	317,221	164	CP157902	4,443,964	1	4,443,964	65.6	4,227	L1	Sensitive
SRR29081006	1,552,074	97	SRR29081047	2,238	113,239	35.6	JBDZWE000000000	4,381,950	10	1,487,978	65.63	4,191	L4	HR-TB
SRR29081044	1,427,562	54	SRR29081117	2,795	297,143	113.6	CP157903	4,435,660	1	4,435,660	65.59	4,222	L1	HR-TB
SRR29081043	183,504	6	SRR29081116	2,983	39,464	18	JBDZWF000000000	4,465,349	6	2,913,614	65.6	4,400	L1	Other
SRR29081042	2,077,602	72	SRR29081115	2,595	398,495	151.7	CP157904	4,445,477	1	4,445,477	65.6	4,233	L1	HR-TB
SRR29081041	2,448,694	83	SRR29081114	2,325	299,648	102	JBEFMN000000000	4,445,435	4	4,438,554	65.61	4,226	L1	Sensitive
SRR29081040	521,152	30	SRR29081113	2,402	264,071	92.6	JBEFMO000000000	4,451,137	5	4,440,512	65.6	4,260	L1	HR-TB
SRR29081039	602,160	34	SRR29081112	2,225	168,478	55	JBDZWG000000000	4,442,392	8	2,914,522	65.6	4,244	L1	Sensitive
SRR29081038	658,400	40	SRR29081111	2,410	122,306	41	JBDZWH000000000	4,441,918	10	3,563,803	65.61	4,258	L1	Sensitive
SRR29081037	1,711,532	72	SRR29081110	2,225	194,212	60.2	JBDZWI000000000	4,444,337	4	2,793,638	65.59	4,235	L1	Sensitive
SRR29081036	749,832	24	SRR29081109	3,385	34,364	17.4	JBEFMP000000000	4,438,716	4	4,431,834	65.6	4,227	L1	Other
SRR29081035	2,215,960	75	SRR29081108	2,347	45,101	16.2	JBDZWJ000000000	4,437,622	8	2,881,951	65.59	4,225	L1	HR-TB
SRR29081033	555,312	33	SRR29081106	2,152	81,008	20.8	JBDZWK000000000	4,455,959	92	326,337	65.56	4,312	L1	Sensitive
SRR29081032	1,135,710	36	SRR29081105	1,751	54,735	13.3	JBDZWL000000000	4,396,282	30	360,450	65.56	4,255	L1	Sensitive
SRR29081031	803,646	42	SRR29081104	1,806	19,567	5.2	JBDZWM000000000	4,401,393	39	313,931	65.57	4,236	L1	Other
SRR29081030	1,099,484	39	SRR29081103	1,904	29,947	8.2	JBDZWN000000000	4,359,366	28	356,360	65.6	4,188	L1	Sensitive
SRR29081029	957,996	54	SRR29081102	1,668	29,321	6.3	JBDZWO000000000	4,415,884	39	329,277	65.58	4,241	L1	Sensitive
SRR29081028	149,904	9	SRR29081101	1,986	39,975	12.2	JBDZWP000000000	4,433,949	337	36,195	65.47	4,403	L1	Other
SRR29081027	283,496	9	SRR29081100	2,049	42,723	12.4	JBDZWQ000000000	4,440,913	303	27,614	65.48	4,458	L1	Sensitive
SRR29081026	1,090,374	52	SRR29081099	1,988	71,808	17.8	JBDZWR000000000	4,430,698	16	2,832,081	65.59	4,212	L1	Sensitive
SRR29081025	414,246	26	SRR29081098	2,599	51,637	16.8	JBDZWS000000000	4,427,717	21	1,055,204	65.58	4,260	L1	Sensitive
SRR29081024	815,048	21	SRR29081097	2,018	32,211	9.6	JBDZWT000000000	4,379,707	64	160,630	65.56	4,276	L1	MDR-TB
SRR29081022	577,082	37	SRR29081095	2,112	47,418	14.1	JBDZWU000000000	4,329,474	23	1,000,153	65.52	4,190	L4	MDR-TB
SRR29081021	1,075,962	69	SRR29081094	3,077	73,735	24.2	JBEHBX000000000	4,448,958	3	4,442,025	65.6	4,257	L1	Sensitive
SRR29081020	472,188	29	SRR29081093	2,718	31,361	11.5	JBDZWV000000000	4,442,829	15	709,344	65.59	4,283	L1	Other
SRR29081019	825,152	27	SRR29081092	2,010	48,938	14.8	JBEFMQ000000000	4,444,498	2	4,443,625	65.59	4,235	L1	Sensitive
SRR29081018	872,894	53	SRR29081091	1,747	25,808	6.5	JBDZWW000000000	4,370,413	40	500,884	65.59	4,219	L1	Sensitive
SRR29081017	869,794	56	SRR29081090	1,808	80,516	20.4	JBDZWX000000000	4,435,513	13	2,247,145	65.58	4,245	L1	Sensitive
SRR29081016	828,568	53	SRR29081089	1,848	126,139	33.3	JBDZWY000000000	4,408,389	21	604,343	65.59	4,236	L1	Other
SRR29081015	1,169,498	75	SRR29081088	6,509	129,249	102.3	CP157905	4,432,855	1	4,432,855	65.6	4,215	L1	HR-TB
SRR29081014	817,158	26	SRR29081087	5,100	21,776	15.3	JBEFMR000000000	4,441,209	3	4,435,681	65.6	4,229	L1	HR-TB
SRR29081013	1,561,300	51	SRR29081086	3,966	20,596	9.9	JBEFMS000000000	4,447,889	3	4,445,558	65.6	4,230	L1	MDR-TB
SRR29081011	1,051,274	34	SRR29081084	3,840	21,566	12.3	JBEFMT000000000	4,429,755	2	4,159,895	65.58	4,255	L1	MDR-TB
SRR29081005	704,570	43	SRR29081083	3,949	74,716	44.3	JBEFMU000000000	4,441,521	3	4,436,000	65.61	4,230	L1	Sensitive
SRR29081004	806,960	48	SRR29081082	2,334	29,461	9	JBDZWZ000000000	4,415,109	27	477,167	65.56	4,245	L1	MDR-TB
SRR29081003	585,444	34	SRR29081081	2,408	41,681	13.6	JBDZXA000000000	4,422,364	19	798,757	65.56	4,261	L1	MDR-TB
SRR29081002	616,668	27	SRR29081080	2,049	27,803	8.2	JBDZXB000000000	4,358,715	32	229,058	65.55	4,205	L4	MDR-TB
SRR29081001	1,311,026	84	SRR29081079	1,955	100,216	28.1	JBDZXC000000000	4,420,504	18	1,116,500	65.58	4,246	L1	MDR-TB
SRR29081000	902,546	45	SRR29081078	1,705	101,026	23.5	JBDZXD000000000	4,375,347	28	453,984	65.58	4,228	L1	MDR-TB
SRR29080999	1,376,902	70	SRR29081077	2,549	30,892	11	JBDZXE000000000	4,277,323	16	950,655	65.52	4,092	L1	Sensitive
SRR29080998	830,112	45	SRR29081076	2,286	50,743	16.5	JBDZXF000000000	4,351,283	19	718,543	65.59	4,164	L4	MDR-TB
SRR29080997	889,292	52	SRR29081075	2,137	72,829	22.1	JBDZXG000000000	4,410,294	21	497,102	65.56	4,245	L1	MDR-TB
SRR29080995	570,890	36	SRR29081073	2,104	66,596	19	JBDZXH000000000	4,412,614	14	615,360	65.58	4,233	L1	RR-TB
SRR29080994	943,830	52	SRR29081072	2,205	30,831	9.3	JBDZXI000000000	4,437,178	20	1,121,572	65.59	4,256	L1	MDR-TB
SRR29080993	843,430	50	SRR29081071	2,136	63,942	17.9	JBDZXJ000000000	4,431,675	25	656,701	65.58	4,263	L1	MDR-TB
SRR29080992	313,840	16	SRR29081070	2,773	25,152	9.7	JBDZXK000000000	4,416,570	149	159,405	65.54	4,291	L1	MDR-TB
SRR29080991	606,728	36	SRR29081069	2,373	23,549	8.1	JBDZXL000000000	4,351,593	29	357,924	65.58	4,197	L4	MDR-TB
SRR29080990	558,162	26	SRR29081068	2,404	20,450	6.8	JBDZXM000000000	4,404,943	43	217,796	65.58	4,248	L1	MDR-TB
SRR29080989	224,216	12	SRR29081067	2,156	33,696	9.7	JBDZXN000000000	4,357,613	335	66,503	65.38	4,367	L1	MDR-TB
SRR29080988	359,490	22	SRR29081066	2,108	13,745	4.3	JBDZXO000000000	4,366,312	64	131,357	65.5	4,267	L1	MDR-TB
SRR29080987	477,928	29	SRR29081065	1,856	30,385	8.7	JBDZXP000000000	4,423,497	37	372,964	65.62	4,259	L1	MDR-TB
SRR29080986	257,572	14	SRR29081064	2,291	40,734	14.1	JBDZXQ000000000	4,394,613	79	151,187	65.61	4,253	L4	MDR-TB
SRR29080984	382,836	23	SRR29081062	2,481	47,322	17.1	JBDZXR000000000	4,462,790	22	459,271	65.59	4,267	L1	RR-TB
SRR29080982	527,722	31	SRR29081060	2,269	78,646	23.7	JBDZXT000000000	4,384,213	13	901,922	65.6	4,229	L1	MDR-TB
SRR29080981	306,870	18	SRR29081059	1,824	34,780	9.6	JBDZXU000000000	4,378,959	62	170,206	65.57	4,290	L1	Other
SRR29080980	236,696	14	SRR29081058	1,804	22,228	6.2	JBDZXV000000000	4,381,548	132	68,187	65.51	4,451	L1	RR-TB
SRR29080979	234,512	13	SRR29081057	1,923	107,864	30.4	JBDZXW000000000	4,448,867	41	486,781	65.57	4,290	L1	Other
SRR29080978	337,474	21	SRR29081056	1,706	31,758	8.3	JBDZXX000000000	4,407,228	77	143,946	65.58	4,309	L1	MDR-TB
SRR29080977	389,366	23	SRR29081055	1,909	36,430	10.7	JBDZXY000000000	4,318,745	422	110,858	65.24	4,324	L1	RR-TB
SRR29080976	271,606	15	SRR29081054	4,346	37,542	24	JBEFMV000000000	4,449,696	4	4,442,821	65.61	4,245	L1	MDR-TB
SRR29081010	510,396	28	SRR29081051	4,559	33,657	19.9	JBDZYA000000000	4,427,785	3	3,674,347	65.6	4,267	L1	MDR-TB
SRR29081009	604,226	36	SRR29081050	4,196	74,966	42.9	JBEFMW000000000	4,375,979	3	4,370,469	65.63	4,166	L4	MDR-TB
SRR29081008	427,414	25	SRR29081049	4,528	110,812	67.4	JBDZYB000000000	4,447,493	5	2,909,863	65.61	4,243	L1	MDR-TB

^*a*^ Lineages (L1, Indo-Oceanic; L4, Euro-American).

^*b*^ Drug resistance profiles based on TB-Profiler (Sensitive, no drug resistance; HR-TB, isoniazid-resistant; Pre-XDR-TB, rifampicin-resistant and any fluoriquinolone or any second-line injectables; XDR, rifampicin-resistant and any fluoroquinolone and any second-line injectables; other, resistance to any drug but none of the said categories).

## Data Availability

The genome sequences have been deposited in GenBank under the BioProject accession number PRJNA1113402. Sequence Read Archives (SRA) and assembly accession numbers are listed in Table 1.
